# Differential involvement of the gamma-synuclein in cognitive abilities on the model of knockout mice

**DOI:** 10.1186/1471-2202-14-53

**Published:** 2013-05-14

**Authors:** Viktor S Kokhan, Gennadiy I Van’kin, Sergey O Bachurin, Inna Yu Shamakina

**Affiliations:** 1 Institute of Physiologically Active Compounds RAS, Chernogolovka, Russia; 2 National Research Center on Addictions, Moscow, Russia

**Keywords:** Gamma-synuclein, Knockout mice, Learning, Memory

## Abstract

**Background:**

Gamma-synuclein is a member of the synuclein family of cytoplasmic, predominantly neuron-specific proteins. Despite numerous evidences for the importance of gamma-synuclein in the control of monoamine homeostasis, cytoskeleton reorganization and chaperone activity, its role in the regulation of cognitive behavior still remain unknown. Our previous study revealed that gamma-synuclein knockout mice are characterized by high habituation scores. Since a number of processes including spatial memory of the environment may affect habituation, in the present study we have carried out behavioral evaluation of spatial and working memory in gamma-synuclein knockout mice.

**Results:**

Inactivation of gamma-synuclein gene led to the improvement of working memory in mice as revealed by passive and active avoidance tests. At the same time behavioral tests, designed to assess spatial learning and memory (Morris water maze and Object location tests), showed no differences between gamma-synuclein knockouts and wild type mice.

**Conclusions:**

These findings indicate that young mice with targeted inactivation of gamma-synuclein gene have improved working memory, but not spatial learning and memory. Our results suggest that gamma-synuclein is directly involved in the regulation of cognitive functions.

## Background

Gamma(γ)-synuclein – is a member of the synuclein family of small cytoplasmic acidic, predominantly neuron-specific proteins [1]. It has been speculated that in the nervous system γ-synuclein is involved in modulation of monoamine transporters [2,3], cytoprotection [4,5], chaperone activity [6], microtubule regulation and microtubule mediated organelle trafficking [7]. However, the exact mechanisms and consequences of this involvement are to be resolved. In cooperation with other members of the family γ-synuclein plays role in regulation of dopaminergic neurotransmission [8,9]. Changes of γ-synuclein expression in peripheral tissues has been linked with metabolic and oncological diseases [10,11].

Our previous study showed that γ-synuclein knockout mice display low levels of anxiety-like behavior, high exploratory activity and enhanced habituation [12]. It is well documented that cognitive abilities correlate with the level of exploratory activity and habituation scores in rodents [13,14]. These data allow to suggest that γ-synuclein may be one of the factors affecting cognitive function. Cognitive dysfunction is one of the most typical characteristics in various neurodegenerative pathologies such as Alzheimer’s and Parkinson’s disease [15,16]. Since γ-synuclein has been implicated in hippocampal axon pathology in Parkinson’s disease [17]. Specific changes of γ-synuclein expression in retina and optical nerve have been reported in Alzheimer’s disease patients as well as in patients with glaucoma [18,19]. Overexpression of γ-synuclein induces neurodegeneration in animal models [20,21]. The first evidence of a possible link between the expression of γ-synuclein and choline acetyltransferase – an important component of cholinergic neurotransmission have been received [22]. At the same time, cholinergic neurotransmission is the key component of cognitive process [23,24]. However, there are no data available on the influence of γ-synuclein on learning and memory.

We hypothesized that γ-synuclein might be involved in some aspects of learning and memory processes in experimental animals. To test this, in the present work, we evaluated learning abilities of mice with target inactivation of γ-synuclein gene in the behavioral tasks that require intact working and spatial memory.

## Results

### Rod suspension test

**Figure 1:**
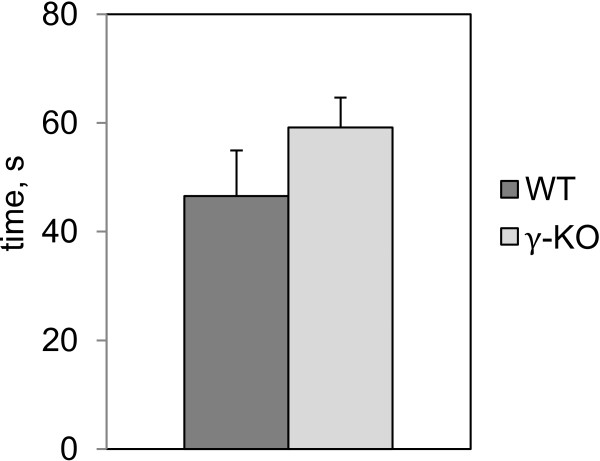
**Rod suspension test.** Bar charts show mean values+S.E.M (WT n=10; γ-КО n=12). No significant differences were detected (Mann–Whitney *U*-test).

On the first stage of the work we examined locomotor abilities of the experimental animals. We assessed the grip strength, which is a critical parameter for swimming task as well as for the other tests which require motor activity. In this test the γ-КО group did not show (p>0.05; Mann–Whitney *U*-test) significant differences in performance from the group of WT mice (Figure 1).

### Passive avoidance test

**Figure 2:**
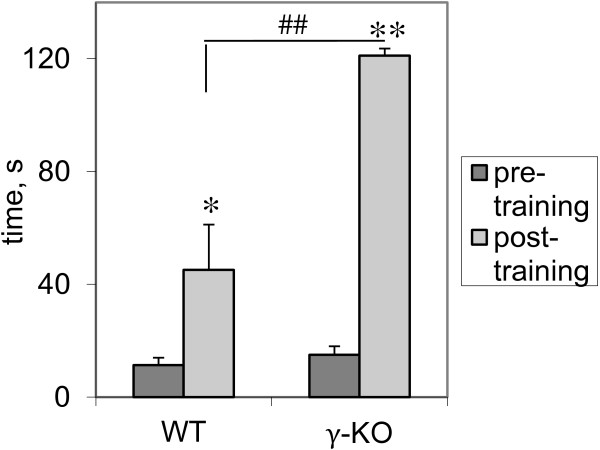
**Passive avoidance test.** Bar charts show mean values+S.E.M before (pre-training) and 24 hours after (post-training) step-down latency training (WT n=7; γ-КО n=9). Asterisks and grids indicate statistically significant differences between the values before and after training within the group (* – p<0.05, ** – p<0.01, Wilcoxon *T*-test; ^##^ – p<0.01, Mann–Whitney *U*-test).

Baseline step down latencies have shown no significant differences between groups at the pre-training stage. In γ-КО mice we observed 7.8-fold (T=0, p=0.008; Wilcoxon *T*-test, hereinafter) increase in step-down latency after training versus 3.9-fold (T=0, p=0.027) increase in WT animals (Figure 2). Thus, γ-KO showed an increase in step-down latency by 168% (U=4.5, p=0.006; Mann–Whitney *U*-test) compared to the WT mice.

### Active avoidance test

**Figure 3:**
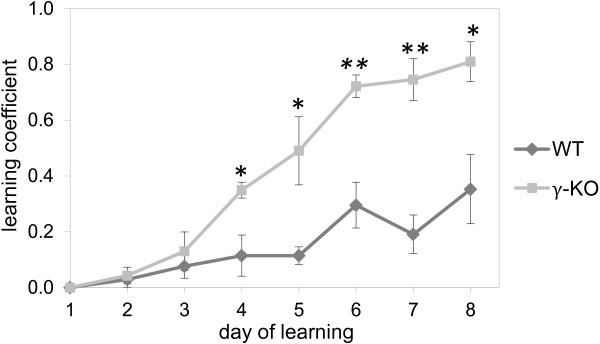
**Active avoidance test.** The curves show mean values±S.E.M (WT n=7; γ-КО n=9). Data are presented as a learning coefficient, being the number of successful attempts (avoidance responses) divided by the total number of transitions. Asterisks indicate statistically significant differences between the groups (* – p<0.05, ** – p<0.01, Mann–Whitney *U*-test).

Mice were trained for 8 days. The results of these experiments expressed as the number of successful avoidance responses over the number of the trials per day are shown in Figure 3. Starting from the 4^th^ day γ-KO displayed better dynamics of learning. γ-KO mice made a significantly greater number of avoidance responses: on Day 4–3 fold (U=8, p=0.037; Mann–Whitney *U*-test, hereinafter), Day 5–4.3-fold (U=7, p=0.015), Day 6–2.4-fold (U=2, p=0.002), Day 7–3.9-fold (U=1.5, p=0.002) and Day 8–2.3-fold (U=6, p=0.01) compared to WT mice.

### Morris water maze

**Figure 4:**
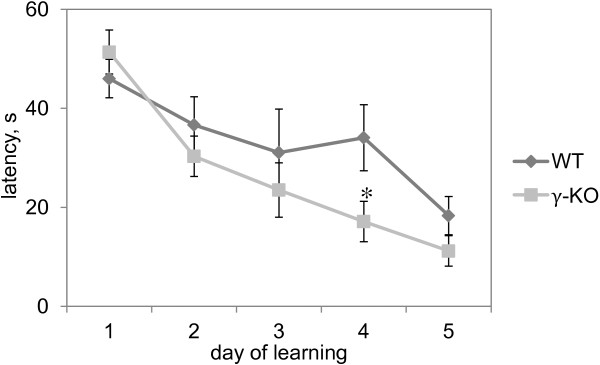
**Morris water maze.** The curves show mean values±S.E.M (WT n=7, γ-КО n=6). Latency (s) is the average (of four trials) time required to reach the platform during each daily session. Asterisks indicate statistically significant differences between the groups (*– p<0.05, Mann–Whitney *U*-test).

To assess spatial learning, mice were tested in the Morris water maze. The results of the test are given in Figure 4. γ-KO and WT mice were not significantly different in all phases of water maze training, except for the training Day 4 when γ-KO mice showed 50% (U=9.5, p=0.03; Mann–Whitney *U*-test) lower escape latencies compared to WT animals.

### Object location test

On day 1 (the acquisition trial) WT- and γ-КО mice time spent equal time investigating both objects (data not shown). On day 2 (test) both WT and γ-КО mice explored the object that was located in a new position for a significantly longer time than the other object (p<0.05; Mann–Whitney *U*-test).

**Figure 5:**
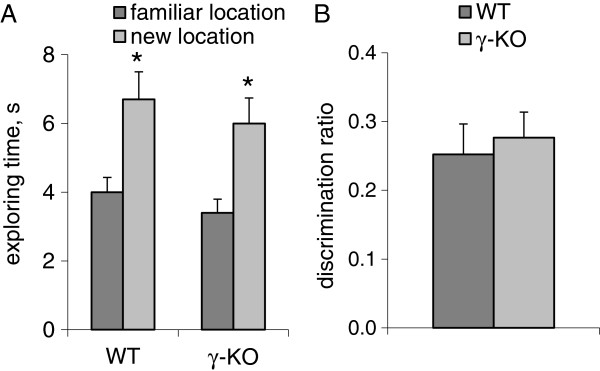
**Object location test.** Bar charts show mean values+S.E.M (WT n=7, γ-КО n=8). **A** – the total exploring time of familiar and displaced object, s. **B** – the discrimination ratio which was calculated as described in the text. Asterisks indicate statistically significant differences between the access value within the group (* – p<0.05, Wilcoxon *T*-test).

γ-КО mice explored the displaced object 1.67 times longer (T=0; p=0.03; Wilcoxon *T*-test, hereinafter), than the object in familiar location. WT mice explored the displaced object 1.76 times longer (T=0; p=0.023), than object in familiar location (Figure 5A). During the test the γ-КО and WT groups showed the same (p>0.05) discrimination ratio: 0.277 and 0.252 for γ-КО and WT groups respectively (Figure 5B).

## Discussion

To analyze locomotor abilities of experimental animals, we estimated the grip strength. Our study found no significant differences of the motor functions in the knockout mice, which is consistent with previously published data obtained in different motor tasks [12]. Intact grip strength by the knockouts suggests that muscle tone is not impaired in these mice.

We have shown that young mice with the target inactivation of γ-synuclein gene are characterized high working-memory capacity, but have no alterations in spatial learning and memory. Our previous study had revealed that behavioral phenotype of γ-KO mice can be characterized by low level of anxiety and enhanced habituation. These data can explain the improvement of learning in the passive and active avoidance models, used in the current investigation.

At the moment molecular and biochemical basis of the memory improvement in passive and active avoidance tests in γ-KO mice is not well understood. Several hypotheses can be assumed. Interestingly, inactivation of the expression of highly homologous to γ-synuclein, α-synuclein protein produces partially opposing phenotype – working and spatial memory impairment in adult mice [25]. However in spite of high homology in the amino acid primary sequence α-synuclein and γ-synuclein proteins differ in their secondary structure: γ-synuclein has an increased α-helical propensity in the amyloid-forming region (NAC-region) [26] which is involved in trafficking of monoamine transporters [3]. We can suggest that variations in the secondary structure are responsible for the opposite effects of α- and γ-synuclein gene knockout on expression of dopamine transporter (DAT) in the brain [27], that can in turn affect their cognitive ability [28]. Moreover, inactivation of the gene expression of α- and γ-synuclein has opposite effects on the emotional status of animals [12,29,30], which may also be reflected in the observed changes in cognitive abilities. It has been shown previously using the same line of knockout mice that inactivation of γ-synuclein gene alone does not affect expression of α-synuclein mRNA or protein in neural tissue [31], therefore our results present a clear effect of γ-synuclein’s inactivation.

It is also possible that in the absence of γ-synuclein a general mechanism of synaptic vesicle turnover and neurotransmitter release are perturbed to the degree that does not noticeably alter animal physiology but affects certain types of behaviour. This idea is consistent with more pronounced changes in neurotransmission observed in α/γ-synuclein double knockout comparing to α-synuclein knockout mice [8] and in triple synuclein knockout comparing to α/β-synuclein double knockout mice [32-34].

γ-Synuclein inactivation did not affect spatial learning in the Morris water maze although γ-synuclein is highly expressed in the brain areas involved in spatial learning [35] and its inactivation was shown to cause developmental deficit in the number of dopamine neurons in the midbrain [27,31] – an essential component for a water maze cued task learning [36]. We also did not reveal alterations in spatial memory of γ-KO mice. The most obvious explanation for this fact can be based on different strategies used for the platform search. “Route navigation” strategy probably allows γ-KO mice to use their high performing working memory and thus compensate the deficient spatial memory which is critical in «locale navigation» strategy [37,38]. This phenomenon as well as enhancement of working memory in γ-KO compared to WT mice needs further investigation.

Thus, our data provide the first evidence that γ-synuclein may be the important component of learning process which primarily based on the functioning of working memory.

## Conclusions

Inactivation of γ-synuclein gene leads to improvement of working memory capacity, but not to change spatial memory and learning. Our data provide the first evidence that γ-synuclein plays an important role in learning process that is primarily based on the functioning of working memory.

## Methods

### Ethical note

The study was approved by the ethical committee of the Institute of Physiologically Active Compounds of RAS, the protocol number 12/12. All methods used were in compliance with the European Communities Council Directive of 24 November 1986 (86/609/EEC).

### Animals

γ-Synuclein homozygous knockout mice on С57BL/6J genetic background were obtained from Cardiff University Transgenic Animal Unit and characterized previously [39]. Briefly, the targeted inactivation of gamma-synuclein transcription was induced through deletion of exons I, II, and III and promoter region of the gene. The control animals with no genome modifications (WT), and the mice with targeted null mutation of the gene encoding γ-synuclein (γ-КО), descended from the common heterozygous ancestors С57BL/6J (Charles River). Genotyping was carried out as described previously [31]. WT and γ-KO cohorts were housed individually in the same conditions on a 12 h light/dark cycle. The mice had free access to food and water and were maintained at the temperature of 19–21°C and 50% humidity. In all the experiments 3-month male mice (20–23 g) were used.

### Behavioral tests

All behavioral tests were performed during the light phase of the cycle. The interval between the behavioral tests was 2 days. Tests were performed in the following order: rod suspension, object location test*,* passive avoidance test, Morris water maze and after that – active avoidance test.

### Rod suspension

Animals are hung by their front paws from a rod 0.7 cm diameter, suspended 20 cm above the ground. The rod is 3 cm in length and terminated on the plastic walls. Latency to drop from the rod (as a criterion of grip strength) is recorded [40]. The experiment was performed during two consecutive days. On the first day the animals were given an opportunity to get acquainted with the test: a mouse was helped to climb up the rod, and in case of the fall in less than two minutes it was put on the rod again (not more than 3 attempts were given). When the mouse managed to stay on the rod for a longer time, it was not taken from there until it falls.. The test was repeated after 24 hours with one attempt given and the latency to drop from the rod was recorded.

### Passive avoidance test

We used a step-down passive avoidance test, which consisted of a Plexiglas cage (25 × 25 × 45 cm) with a grid floor and a platform (6 × 6 × 1.2 cm) in the center. During the training session, mice were placed on the platform and covered with a beaker. After 35 seconds the glass was removed and the latency to step down with all four paws was measured. Immediately after stepping down on the grid, the animals received an electric shock (80V sinusoidal voltage). Retention test sessions were carried out 24 h after training. Each mouse was placed on the platform and the step-down latency was measured in the absence of electric foot shocks. Step-down latency was used as a measure of memory retention [41]. A cut-off time of 120 sec was set.

### Active avoidance test

The test was performed as described before [25]. Two-way shuttle avoidance begins when a mouse is placed into one of the two equal compartments of the shuttle-box. The compartments have independently controlled electrified grid floors and illumination. Each trial is separated by 10 sec and consists of a 6-sec light-on that is followed by unipolar square electric impulses (30 V, 100 Hz) through the grid in the compartment where the mouse is located. Both the light and foot shock are terminated when the animal crosses to the alternate chamber. We used 5 trials a day during the Days 1–2 and 15 trials – during the Days 3–5. Successful avoidances consist of the trials where the mouse crosses to the adjacent chamber following the onset of the light, but before the foot shock.

### Morris water maze test

A standard Morris water maze test was carried out in a pool (70 cm in diameter) of water at 22–23°C. An escape platform 6 cm in diameter was placed 0.5 cm below the surface of the water in the middle of one of the four quadrants of the pool. The platform and pool was black with anti-glare coating, which together with shadowless lamp lighting creates the effect of an invisible platform which is hidden under water. As visual cues we used 3 illuminated paper-figures fixed on the walls. Mice were placed into the tank, facing the wall of the pool, and were allowed to navigate the pool in search of the escape platform for a maximum of 60 s. If an animal failed to locate the platform within 60 s during the first training day, it was guided to the platform by the experimenter. The start points used for each trial varied. The time to reach the escape platform was recorded, and the animals were permitted 30 s to rest on the platform before removal from the tank [42]. Mice were tested during 5 days on 4 daily trials which were then averaged.

### Object location test

The test was performed as described before [43] with some modifications. Mice were placed into a gray Plexiglas open-field box (40 cm wide×35 cm deep×16 cm high). Brown glass identical vials (5 cm in height×3 cm in diameter) were used as objects. The illumination was 60 lux in the center of box. The acquisition trial: mice were introduced to two identical objects located within the open field arena for 5 min. After a delay of 24 hours mice were introduced to the same arena but one of the objects was moved to a new location. The time the mouse spent in exploring each object was recorded for 5 min. The object-location discrimination ratio was used as a criterion of spatial memory [43,44] and calculated as (T1-T2)/(T1+T2), where T1 - the time spent by the animal exploring the new-located object, T2 – the old-located object.

### Statistical analysis

Standard data processing was performed with Statistica 8 software (StatSoft Inc., USA). Depending on normality verified using Shapiro-Wilk’s W-test, either *t*-test for dependent or independent samples, or non-parametric Mann–Whitney *U*-test for independent groups and Wilcoxon *T*-test for dependent samples were used.

## Abbreviations

γ-КО: γ-synuclein knockout mice; WT: Wild type mice with no genome modifications.

## Authors’ contributions

Conceived and designed the experiments: VSK, SOB, IYuS. Performed the experiments: VSK, GIV. Analyzed the data: VSK. Contributed materials/analysis tools: GIV, IYuS. Wrote the paper: VSK, IYuS. All authors read and approved the final manuscript.
